# Challenging Management of Post-transcatheter Aortic Valve Implantation (TAVI) Infective Endocarditis With Neurological and Spinal Complications in an Elderly Patient: A Case Report

**DOI:** 10.7759/cureus.91958

**Published:** 2025-09-10

**Authors:** Shuhei Yamazaki, Tatsuya Tanaka, Anh Tran Hue, Akira Saito, Akira Matsuno

**Affiliations:** 1 Department of Neurosurgery, International University of Health and Welfare, Narita Hospital, Narita, JPN

**Keywords:** cerebral embolism, elderly patients, infective endocarditis, minimally invasive, percutaneous pedicle screws, pyogenic, spine stabilization, spondylitis, streptococcus sanguinis, transcatheter aortic valve implantation

## Abstract

Infective endocarditis (IE) following transcatheter aortic valve implantation (TAVI) is a rare but life-threatening complication, particularly in elderly patients with multiple comorbidities. We describe a case of an 84-year-old woman who developed IE three years after TAVI. The patient initially presented with cerebral embolism and was found to have *Streptococcus sanguinis* bacteremia. Although initial clinical and imaging findings met only minor Duke criteria, transesophageal echocardiography (TEE) later confirmed a definitive diagnosis by identifying a mobile vegetation measuring 11.2 mm on the prosthetic valve. During hospitalization, the patient developed pyogenic spondylitis, likely secondary to hematogenous spread. Conservative management with antibiotics was initiated; however, persistent back pain and impaired mobility necessitated posterior fixation using percutaneous pedicle screw (PPS). Given her advanced age and underlying liver cirrhosis, surgical valve replacement was considered high-risk and thus, not performed. The patient demonstrated significant clinical improvement with a combination of antimicrobial therapy and minimally invasive spinal surgery. She was subsequently discharged on oral antibiotics with regained functional independence. This case highlights the complexities of diagnosis and management of post-TAVI IE with secondary spinal involvement in geriatric populations.

## Introduction

Aortic stenosis (AS) is one of the most prevalent valvular heart diseases among elderly individuals. When the disease advances, AS can manifest with exertional dyspnea, angina, syncope, and a markedly poor prognosis. While surgical aortic valve replacement (SAVR) has traditionally been the standard treatment for severe AS, transcatheter aortic valve implantation (TAVI) has emerged over the past decade as a less invasive alternative, particularly suited for elderly patients or those with significant operative risk and multiple comorbidities [1].

With the expanding use of TAVI, cases of infective endocarditis (IE) following TAVI (post-TAVI IE) have been increasingly reported, with an incidence ranging from 0.6% to 3.4%, and some studies suggest that it may exceed that seen with SAVR [2,3]. Post-TAVI IE often presents in frail, elderly patients and is associated with nonspecific clinical features that complicate diagnosis [4-10]. Severe complications, such as heart failure, annular abscesses, periprosthetic dehiscence, and systemic embolization (particularly to the central nervous system), are frequent and can significantly worsen prognosis [4-10].

Hematogenous dissemination may also result in secondary spinal infections, including vertebral osteomyelitis and discitis, which can further worsen patients' mobility and quality of life (QOL) [11-13]. These spinal complications can compromise treatment, especially in patients who have a high risk for surgery.

In this report, we present a case of an 84-year-old patient who developed post-TAVI IE complicated by cerebral embolism and pyogenic spondylitis. Despite fulfilling surgical criteria, the patient was managed conservatively due to her comorbidities. Notably, minimally invasive spine stabilization was performed to preserve her mobility. This case offers a valuable understanding of the diagnosis and management of post-TAVI IE in elderly patients with multiple comorbidities.

## Case presentation

**Figure 1:**
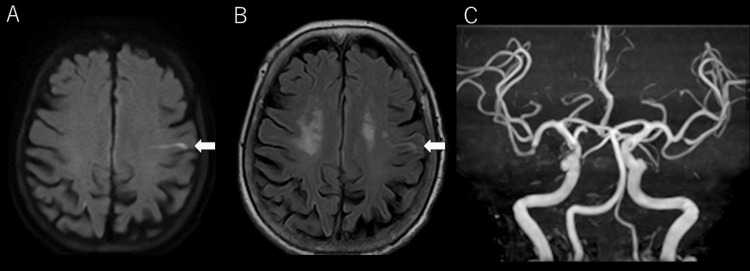
Brain Magnetic Resonance Imaging (MRI) and Magnetic Resonance Angiography (MRA) on Admission Diffusion-weighted imaging (DWI) (A) and fluid-attenuated inversion recovery (FLAIR) (B) show a hyperintense lesion in the left frontal lobe (white arrows), consistent with acute cerebral infarction. MRA (C) shows no evidence of major intracranial arterial occlusion.

An 84-year-old woman with a medical history of severe AS (treated with TAVI at Hospital A three years prior), atrial fibrillation (catheter ablation performed at Hospital B two years prior), hypertension, type 2 diabetes mellitus, and liver cirrhosis presented to our emergency department with a new-onset slurred speech upon awakening. Her symptoms persisted throughout the day, prompting hospital evaluation. On arrival, she was alert and conscious with a Glasgow Coma Scale (GCS) score of 15, and her National Institutes of Health Stroke Scale (NIHSS) score is 2, with isolated dysarthria without focal motor or sensory deficits. Electrocardiography (ECG) showed a regular RR interval without atrial fibrillation. Brain magnetic resonance imaging (MRI) revealed a hyperintense lesion in the left frontal lobe on diffusion-weighted imaging (DWI), with corresponding signal changes on fluid-attenuated inversion recovery (FLAIR), consistent with an acute cerebral infarction. Magnetic resonance angiography (MRA) showed no evidence of major intracranial arterial occlusion (Figure 1).

A diagnosis of cerebral embolism was made, and the patient was treated with continued apixaban (2.5 mg twice daily), which she had been taking previously, in addition to intravenous edaravone (30 mg twice daily).

**Figure 2:**
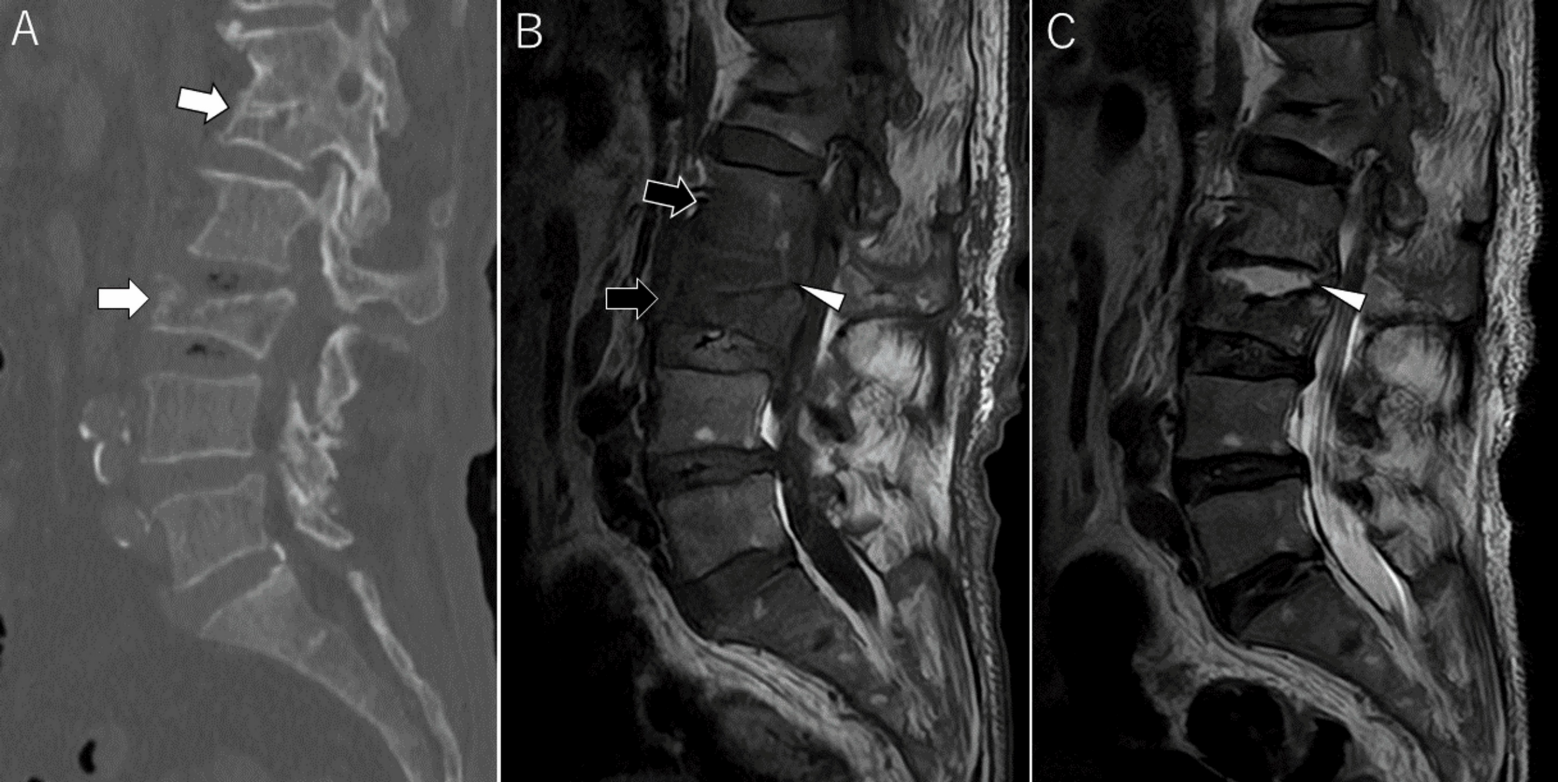
Lumbar Spine Computed Tomography (CT) and Magnetic Resonance Imaging (MRI) Lumbar CT (A) reveals compression fractures of the first and third lumbar vertebral bodies (white arrows). T1-weighted imaging (T1WI) (B) shows hypointensity in the second and third lumbar vertebral bodies (black arrows) and irregular margins with low signal intensity in the L2/3 intervertebral disc. T2-weighted imaging (T2WI) (C) shows hyperintensity in the L2/3 disc (arrowhead). These findings are suggestive of pyogenic spondylitis.

On hospital day two, she developed a fever of 38.1°C with preserved appetite and no signs of skin lesions (e.g., Janeway lesions, Osler’s nodes) or ocular findings (e.g., conjunctival hemorrhages, Roth spots). Blood cultures were obtained, and one of two sets grew *Streptococcus sanguinis*. She also complained of new-onset lower back pain with spinal tenderness. Lumbar computed tomography (CT) revealed compression fractures at L1 and L3 (Figure 2A), raising concern for IE and secondary pyogenic spondylitis. Empirical antibiotic therapy was initiated. Repeat blood cultures again isolated *S. sanguinis*, fulfilling three minor Duke criteria: fever >38°C, embolic stroke, and positive blood cultures. Transthoracic echocardiography (TTE) showed no vegetations or perivalvular complications. As only three minor Duke criteria were met, a definitive diagnosis of IE could not be confirmed. Given her history of atrial fibrillation, cerebral embolism from paroxysmal atrial fibrillation remained a differential diagnosis, and apixaban was continued alongside antibiotics. Serial MRIs and MRAs were performed to monitor for any new-onset cerebral embolism or formation of infectious cerebral aneurysms (mycotic aneurysm).

An oral examination revealed generalized gingivitis and a dental caries at the right mandibular canine, suggesting the oral cavity as the likely source of bacteremia. Subsequently, the patient received dental treatment, including removal of the infected tooth and local oral hygiene management.

By hospital day six, the patient became afebrile, and blood cultures turned negative. On hospital day eight, lumbar MRI showed T2 hyperintensity and T1 hypointensity in the L2-L3 intervertebral disc with destructive changes in the L3 vertebral body, consistent with pyogenic spondylitis (Figures 2B-2C).

**Figure 3:**
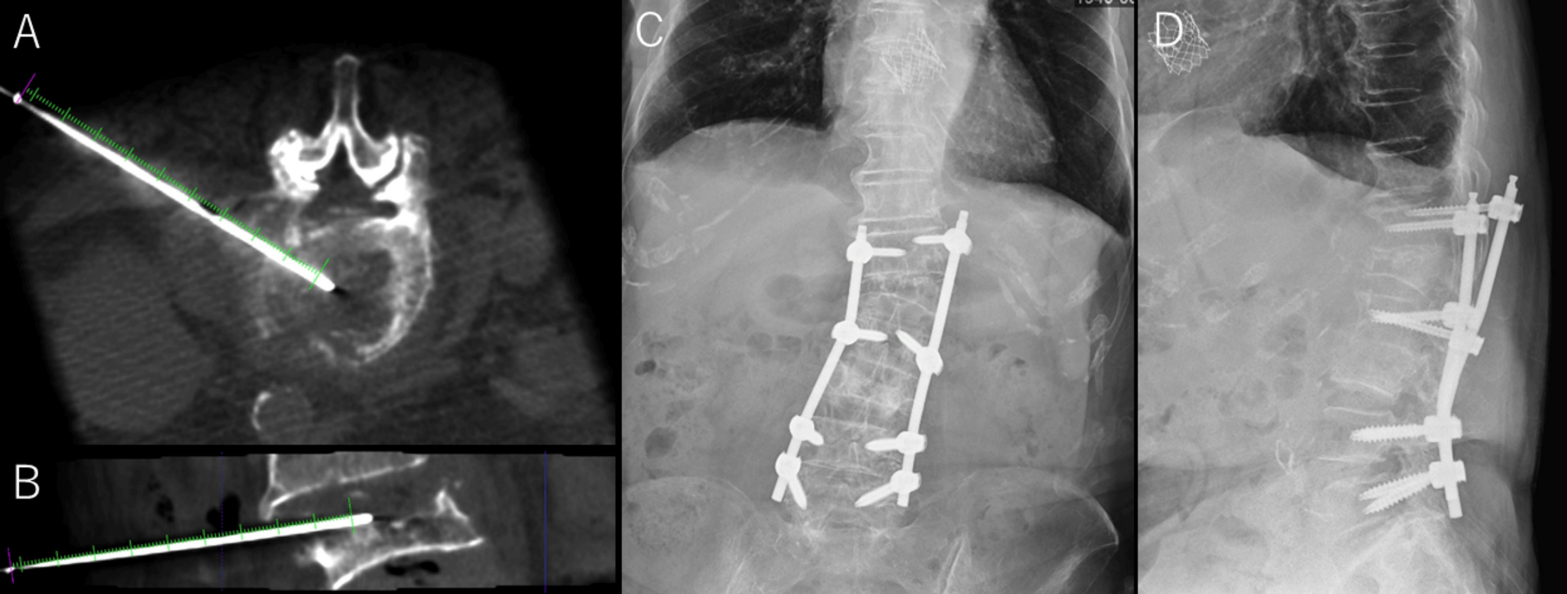
Surgical Intervention for Pyogenic Spondylitis CT-guided disc aspiration was performed at the L2/3 level. Axial (A) and sagittal (B) images confirm the needle reaching the intervertebral disc space. Postoperative radiographs demonstrate posterior fixation using percutaneous pedicle screws at the twelfth thoracic, second, fourth, and fifth lumbar vertebrae (C: anteroposterior view, D: lateral view).

Despite antibiotic and analgesic therapy, her back pain and limited mobility persisted. On hospital day 19, CT-guided disc aspiration was performed at L2-L3 for diagnostic purposes and to reduce intradiscal pressure (Figures 3A-3B). The aspirated fluid was nonpurulent, and cultures were negative. Initial conservative treatment with a rigid corset and analgesia failed to alleviate her back pain, and her ADL deteriorated. On hospital day 26, percutaneous pedicle screws (PPS) were placed from T12 to L2, L4, and L5 to stabilize the affected segments (Figures 3C-3D).

**Figure 4:**
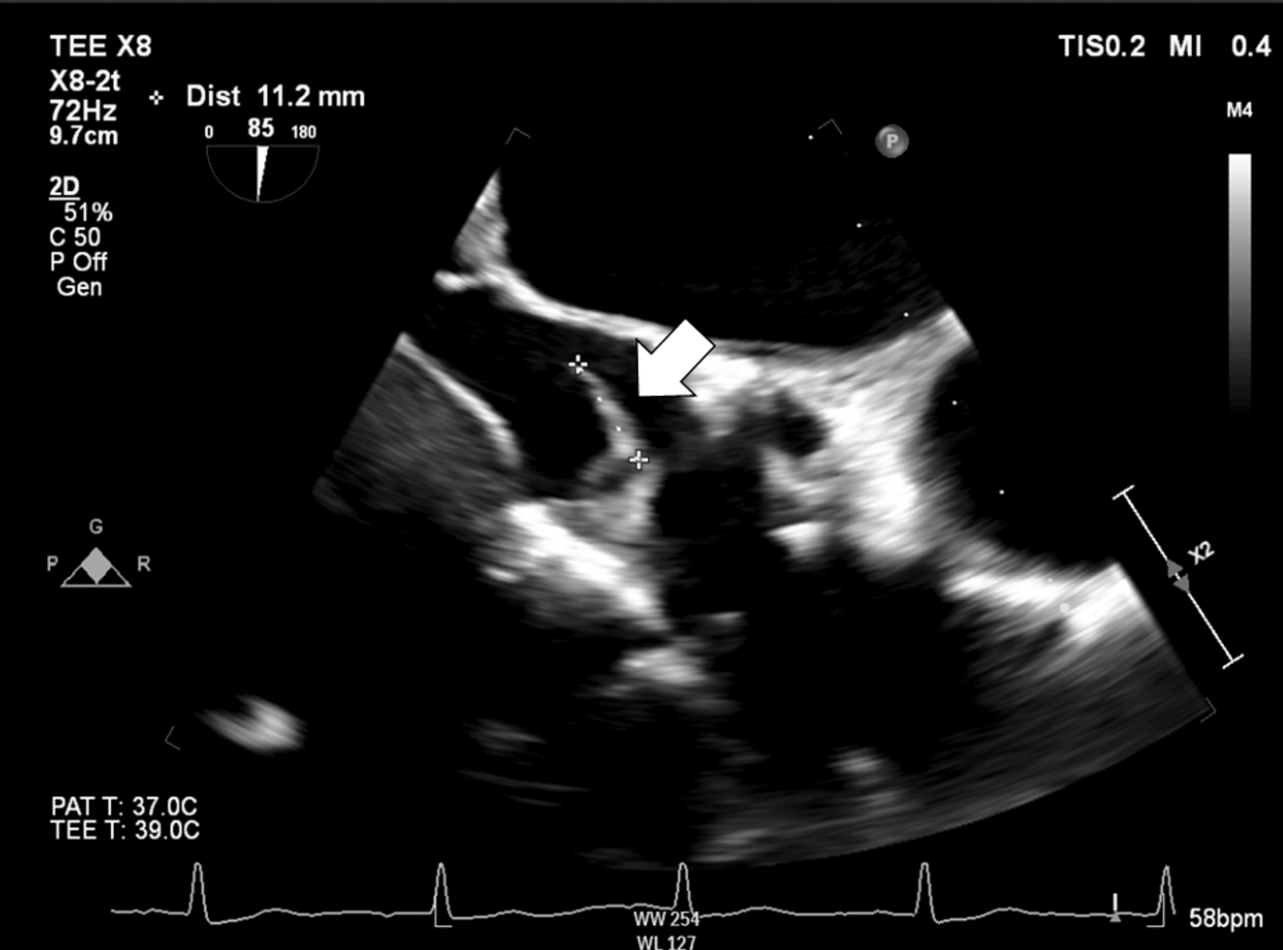
Transesophageal Echocardiography Findings A mobile, linear structure measuring 11.2 mm is observed on the transcatheter aortic valve prosthesis (white arrow), consistent with vegetation due to infective endocarditis.

Postoperatively, her back pain improved, and rehabilitation was continued while wearing the corset. On hospital day 32, transesophageal echocardiography (TEE) identified a mobile vegetation measuring 11.2 mm on the TAVI prosthesis (Figure 4), therefore fulfilling one major and three minor Duke criteria, confirming the diagnosis of definite IE.

Despite the high risk of recurrent embolism, surgical resection was not pursued due to her advanced age and compromised systemic condition, including liver cirrhosis. Conservative treatment was continued. Follow-up TTE was performed during the clinical course; however, no repeat TEE was undertaken due to the patient’s high surgical risk and the pre-established decision to avoid open-heart surgery regardless of imaging findings.

Regarding antimicrobial therapy, ceftriaxone (2 g/day) was initiated on hospital day two, but switched to vancomycin on day three. As the patient's C-reactive protein (CRP) levels continued to rise, suggesting inadequate infection control, combination therapy with ceftriaxone (2 g/day) and ampicillin (2 g/day) was initiated. On hospital day 40, the patient developed a generalized maculopapular rash. Suspecting a drug-induced eruption, both ceftriaxone and ampicillin were discontinued, and clindamycin (1.2 g/day) was introduced as an alternative. However, on hospital day 47, a second rash occurred, leading to discontinuation of clindamycin. Vancomycin was reintroduced thereafter and was well tolerated without adverse reactions. Blood cultures remained negative throughout. Eventually, on hospital day 78, the antibiotic regimen was switched to oral minocycline (200 mg/day). The isolated *S. sanguinis* strain was sensitive to all tested antimicrobials, with minimum inhibitory concentration (MIC) values falling within the susceptible range based on Clinical and Laboratory Standards Institute criteria. By hospital day 91, the patient was ambulatory with the corset and was discharged home. She continues to receive oral antibiotic therapy as an outpatient with improved ADL and no evidence of recurrent infection. The total duration of antimicrobial therapy was planned to continue until inflammatory markers such as CRP and erythrocyte sedimentation rate returned to normal, in accordance with the resolution of pyogenic spondylodiscitis.

**Figure 5:**
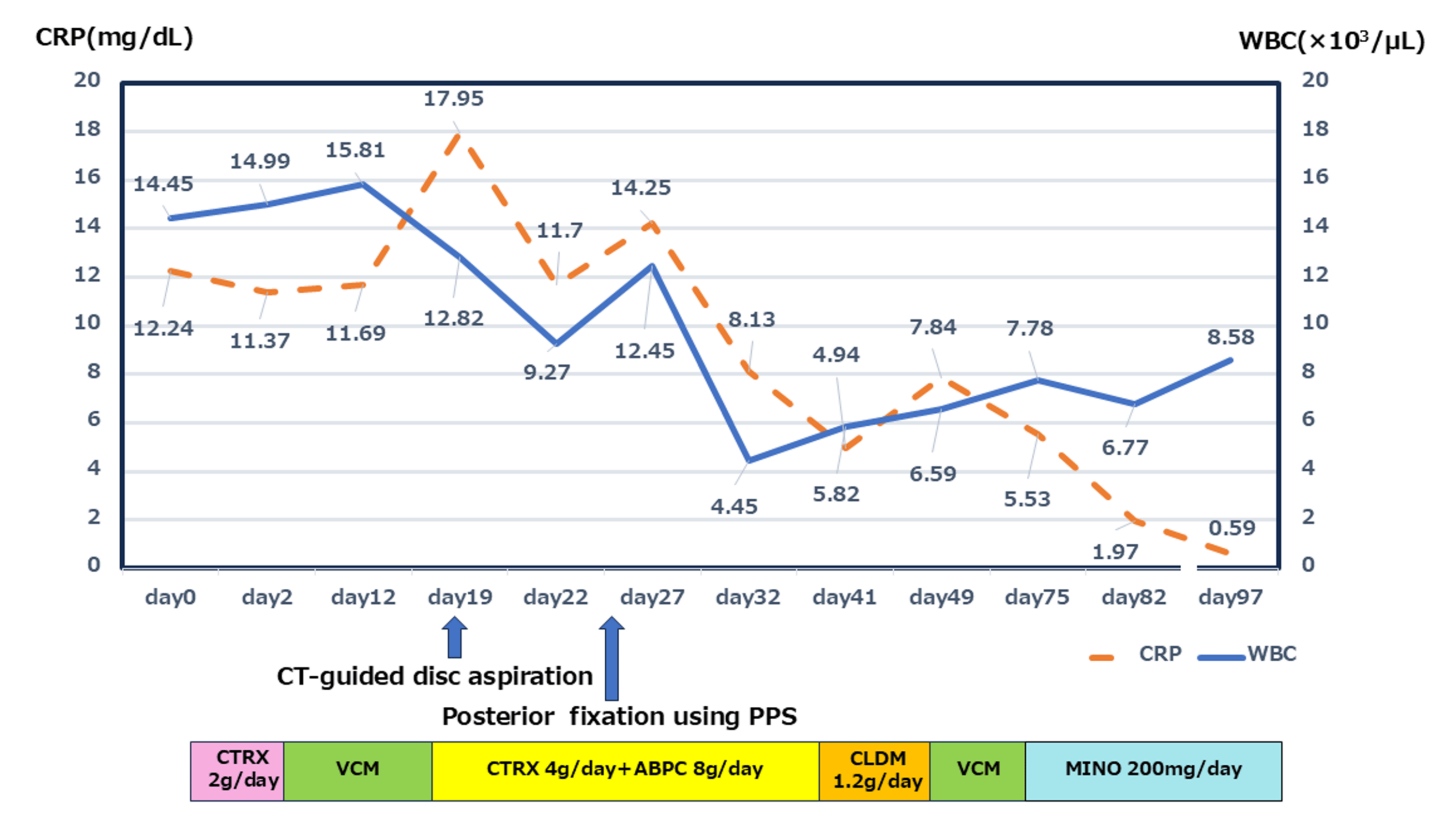
Clinical Course of Antibiotic Therapy and Inflammatory Markers Trends in C-reactive protein (CRP, dashed line; mg/dL) and white blood cell count (WBC, solid line; × 10³/μL) are shown over time. The patient underwent CT-guided disc aspiration on hospital day 19 and posterior spinal fixation using percutaneous pedicle screws (PPS) on day 26. The antibiotic regimen changed according to the clinical response and suspected drug eruptions, as shown in the timeline below the graph. CTRX, ceftriaxone; VCM, vancomycin; ABPC, ampicillin; CLDM, clindamycin; MINO, minocycline; PPS, percutaneous pedicle screws

The clinical course, including antibiotic regimen and changes in white blood cell count and CRP, is shown in Figure 5.

## Discussion

This case describes an 84-year-old female who developed late-onset IE post-TAVI, due to *S. sanguinis*, and complicated by cerebral embolism and pyogenic spondylitis. *S. sanguinis* is a commensal oral bacterium and is known to cause IE in patients with poor oral hygiene, such as gingivitis and dental caries. In this patient, her underlying immunosuppressive conditions, including diabetes mellitus and liver cirrhosis, are likely predisposing her to the development of infection.

The diagnosis of IE is based on the modified Duke criteria, which categorizes cases as “definite,” “possible,” or “rejected” [14]. Although the modified Duke criteria are sensitive for diagnosing native valve IE, recent reports have shown that their sensitivity drops to approximately 50% in cases of TAVI-prosthetic valve endocarditis (PVE), compared to 100% sensitivity using the criteria by the European Society of Cardiology (ESC) [15]. This discrepancy may be explained by the inclusion of multimodal imaging in the ESC. Transcatheter valves contain a substantial amount of metal, which can hinder the visualization of vegetations and limit the diagnostic utility of both TTE and TEE [16]. In such cases, alternative imaging modalities, such as 18-FDG PET/CT, can be obtained [17]. In our case, the patient initially fulfilled only three minor Duke criteria - fever >38°C, positive blood cultures for *S. sanguinis*, and embolic stroke - with TTE showing no vegetations or perivalvular complications. Therefore, the diagnosis was provisionally classified as "possible infective endocarditis" based on the modified Duke criteria. A definitive diagnosis was later confirmed when TEE revealed a mobile vegetation measuring 11.2 mm on the prosthetic valve, fulfilling one major and three minor criteria. This emphasizes the importance of TEE, especially in PVE, where TTE sensitivity may be limited.

Current guidelines from the ESC and the American Heart Association/American College of Cardiology (AHA/ACC) recommend surgical intervention in patients with IE who develop (1) heart failure due to severe valvular dysfunction, (2) uncontrolled infection despite antibiotics (e.g., abscess, persistent bacteremia, or multidrug resistance), and (3) large vegetations (>10 mm) with embolic risks [18,19]. In prosthetic valve IE, early surgery is favored due to the increased incidence of perivalvular extension and annular complications [18,19]. TAVI-associated IE generally follows similar surgical criteria.

Our patient met surgical indications, with evidence of embolic stroke and a large mobile vegetation. However, due to advanced age and comorbidities, the risks associated with open-heart surgery were deemed excessive, and conservative treatment was chosen. Such decisions are not uncommon; studies have shown that, among patients who fulfill surgical criteria, only 3.7-16.4% undergo surgery [7,9,10]. Frailty, high Society of Thoracic Surgeons risk scores, severe heart failure, sepsis, and perivalvular abscess are frequently cited as barriers to surgical intervention [7,9,10].

Outcomes with conservative therapy are generally poor. The one-year mortality rate is reported to be 40-50% and increases to 73.7% in patients who meet surgical criteria but do not undergo surgery [8,20]. In contrast, a one-year mortality rate of only 4.2% has been reported in patients who receive surgical treatment [8,20]. However, other studies have reported no significant survival benefits, underscoring the need for individualized decision-making based on patient-specific conditions.

Another feature of this case was the development of pyogenic spondylitis secondary to hematogenous spread. Spinal infections are a rare complication, but several reports have described similar pyogenic spondylitis following TAVI. Sherlock et al. reported a case of lumbar discitis and osteomyelitis caused by *Erysipelothrix rhusiopathiae*, which was treated conservatively with antibiotics [12], while Glueck et al. described a case of cervical discitis caused by *Enterococcus faecalis* five weeks after TAVI [13]. Similar to these cases, we initially chose conservative treatment with antibiotics and a rigid lumbar brace; however, our patient experienced persistent pain and deteriorating mobility. Therefore, posterior spinal stabilization using PPS was performed. PPS offers the advantages of minimal invasiveness, reduced blood loss, and lower perioperative stress, which are critical in elderly patients with multiple comorbidities. While debridement is traditionally part of surgical management in pyogenic spondylitis, PPS without debridement has been reported to achieve good outcomes in select cases [21].

Although the onset of infective endocarditis in this case occurred three years after TAVI, which may be considered a relatively late presentation, PVE is known to occur even several years post-implantation [7,20]. TAVI-related endocarditis can develop at any time following the procedure, particularly in elderly or immunocompromised patients. Chronic endothelial trauma, persistent bacteremia from dental or gastrointestinal sources, and biofilm formation on the prosthetic surface may all contribute to late-onset infection. *S. sanguinis*, an oral commensal bacterium, was identified as the causative organism in this case, and the patient had poor oral hygiene, which likely served as the source of bacteremia. Several studies have indicated that late-onset PVE (>1 year after TAVI) is not uncommon and accounts for nearly half of all TAVI-IE cases, underscoring the need for long-term vigilance, even years after implantation [7,20].

## Conclusions

This case highlights several important clinical considerations. First, post-TAVI IE may present with atypical features and may be difficult to diagnose using standard tools alone; therefore, a high index of suspicion and early use of TEE or advanced imaging is crucial. Second, although surgical intervention is generally favored in patients with large vegetations or embolic events, individualized treatment planning based on age, comorbidities, and operative risk is essential. Finally, this case contributes to the growing literature on secondary spinal infections in post-TAVI IE and demonstrates that, even in patients ineligible for open surgery, favorable outcomes can be achieved through tailored conservative and minimally invasive approaches.
